# Routine Immunization Service Delivery Through the Basic Package of Health Services Program in Afghanistan: Gaps, Challenges, and Opportunities

**DOI:** 10.1093/infdis/jiw549

**Published:** 2017-06-30

**Authors:** Chukwuma Mbaeyi, Noor Shah Kamawal, Kimberly A. Porter, Adam Khan Azizi, Iftekhar Sadaat, Stephen Hadler, Derek Ehrhardt

**Affiliations:** 1 US Centers for Disease Control and Prevention, Atlanta, Georgia;; 2 Afghanistan Ministry of Public Health, Kabul

**Keywords:** Routine Immunization, BPHS, Afghanistan, Survey, Health Service Delivery, Polio Eradication.

## Abstract

**Background.:**

The Basic Package of Health Services (BPHS) program has increased access to immunization services for children living in rural Afghanistan. However, multiple surveys have indicated persistent immunization coverage gaps. Hence, to identify gaps in implementation, an assessment of the BPHS program was undertaken, with specific focus on the routine immunization (RI) component.

**Methods.:**

A cross-sectional survey was conducted in 2014 on a representative sample drawn from a sampling frame of 1858 BPHS health facilities. Basic descriptive analysis was performed, capturing general characteristics of survey respondents and assessing specific RI components, and χ^2^ tests were used to evaluate possible differences in service delivery by type of health facility.

**Results.:**

Of 447 survey respondents, 27% were health subcenters (HSCs), 30% were basic health centers, 32% were comprehensive health centers, and 12% were district hospitals. Eighty-seven percent of all respondents offered RI services, though only 61% of HSCs did so. Compared with other facility types, HSCs were less likely to have adequate stock of vaccines, essential cold-chain equipment, or proper documentation of vaccination activities.

**Conclusions.:**

There is an urgent need to address manpower and infrastructural deficits in RI service delivery through the BPHS program, especially at the HSC level.

In 2003, the government of Afghanistan introduced the Basic Package of Health Services (BPHS) program [1]. BPHS was established to improve access to healthcare services in rural areas, which account for >70% of Afghanistan’s population of >32 million persons [2]. Those living in these areas are mostly engaged in agricultural and vocational work, with lower literacy rates and limited access to health services. BPHS comprises several key elements, including maternal and newborn care, child health and immunization, and communicable disease control [1, 3]. These services are provided through different tiers of the primary health sector, ranging from small health posts catering to approximately 100–150 families to district hospitals (DHs), which serve populations of tens of thousands persons. Health services administered through BPHS are provided on a graduated scale, with the higher tiers of health facilities providing a more comprehensive package of services compared with smaller health facilities. 

Routine assessments of BPHS since inception, using instruments such as the Afghan Health Survey and Multiple Indicator Cluster Survey, indicate that the program has contributed to reducing infant mortality and increasing immunization coverage [4–7]. However, although BPHS has increased overall access to immunization services in rural areas, significant gaps remain, as evidenced by the findings of a 2015 Afghanistan Demographic and Health Survey showing that none of the country’s 34 provinces had proportions of fully immunized children up to 80% [8]. Immunization coverage among children in rural areas was also significantly lower than in urban areas. Routine immunization (RI) constitutes one of 4 key strategies for polio eradication [9], and Afghanistan, a polio-endemic country [10], is a priority country earmarked for RI strengthening by the Global Polio Eradication Initiative. Hence, to identify gaps in its implementation and ensure prompt remediation of program deficiencies, a comprehensive assessment of the BPHS program was undertaken, with specific focus given to its RI component. In this report, we present the findings of the assessment.

## METHODS

### Survey Design

A cross-sectional survey was conducted in 2014 on a representative sample of health facilities drawn from the Health Management Information System database. The study protocol was developed by a consortium of partners from the Afghan Ministry of Public Health, the US Centers for Disease Control and Prevention (CDC), the World Health Organization, and Apex Consulting. A structured questionnaire was designed based on the study objectives to assess different RI components as implemented under BPHS, including vaccine availability, cold-chain management, service delivery, microplanning, and social mobilization, as well as documentation. Ethical review and approval of the study protocol was undertaken by the Afghanistan National Public Health Institute.

### Sampling, Data Collection, and Statistical Analysis

From a sampling frame of 1858 BPHS health facilities providing RI services in rural Afghanistan, 490 facilities were selected proportionate to the size of each facility. The calculation was based on an expected proportion of 50% of facilities meeting a dichotomous indicator, desired precision of ±7% and 95% probability of achieving that precision. Data were then collected from these facilities using structured questionnaires administered to the designated health officer in charge of the facility. Questionnaires were field tested and corrected before actual data collection and were translated into the local languages (ie, Pashto and Dari), for ease of administration in specific areas. Data from completed surveys were then entered into EpiData software (EpiData, version 3.1) and subsequently exported to SAS 9.3 statistical software (SAS Institute), which was used for statistical analysis. 

Basic descriptive analysis was performed on variables capturing general characteristics of survey respondents and also for responses to a majority of questions. Travel distances and times were assessed from the provincial center to ensure standardization of responses. Statistical means with confidence intervals (CIs) were computed for several continuous and discrete variables. Medians were sometimes reported in lieu of means, and χ^2^ tests were used to evaluate RI components for possible differences due to a specific explanatory variable for binary and categorical responses that might have varied by an explanatory variable. Given its significance to the quality of service delivery, the type/level of the health facility was used as a primary unit of analysis (ie, as an explanatory variable) to identify disparities in the quality of RI services at the different levels of service delivery and establish the principal drivers of gaps and deficiencies in the system. 

For the purpose of this survey, health subcenters (HSCs) represented the lowest level of service delivery, with higher levels of services offered by basic health centers (BHCs) and comprehensive health centers (CHCs), and DHs represented the highest level of service delivery among survey participants. HSCs, as lowest level of service delivery, formed the baseline group for comparison with other facility types. Hence, results were commonly reported by comparing the level of a particular characteristic/service at HSCs to levels in other facility types, using the lowest percentage among the other 3 facility types as a threshold for comparison with HSCs. This was done for ease of presentation of results. Statistical significance was set at a *P* value cutoff of <.05. *P* values, where reported, should be interpreted with caution, given the small numbers of facilities (<20) in several subcategories, resulting in unstable estimates.

## RESULTS

### Facility Description and General Characteristics

A total of 447 health facilities participated in the survey, representing 24% of 1858 eligible health facilities and 91% of the planned enrollment of 490 facilities (Table 1). Participating facilities were sampled from all 34 provinces. Of survey respondents, 27% were HSCs, 30% were BHCs, 32% were CHCs, and 12% were DHs. Health facilities offered patient care services for a daily mean of 6.48 (95% CI, 6.31–6.64) hours, and the mean distance from the provincial center to a health facility was 26.67 (24.04–29.30) km. Mean distances ranged from 23.67 (95% CI, 16.39–30.94) km for DHs to 28.48 (23.65–33.30) km for BHCs. The mean travel time from the provincial center to a health facility was 3.15 (95% CI, 2.44–3.86) hours, with mean travel times ranging from 2.37 (1.75–2.99) hours for DHs to 3.80 (2.38–5.22) hours for HSCs. Eighty-three percent of participating facilities had reception/registration rooms, but only 75% had waiting rooms for patients. An even smaller proportion of facilities (66%) reported having a separate waiting room for women. Only 71% had heating available in the patient areas during winter. Compared with other types of health facilities, HSCs were less likely to report having a registration room (71% vs >80%; *P* < .001), separate waiting room for women (54% vs >67%; *P* < .006), or heating in patient areas during winter (57% vs >74%; *P* < .001). 

**Table 1:** General Characteristics of Respondents in a Survey of Health Facilities Providing the Basic Package of Health Services in Afghanistan, 2014

Variable	HSCs (n = 121)	BHCs (n = 133)	CHCs (n = 141)	DHs (n = 52)	Overall (N = 447)	*P* Value^a^
Daily duration of patient care, mean, h	6.41	6.51	6.31	7.00	6.48	…
Travel time from provincial center, mean, h	3.80	2.78	3.23	2.37	3.15	…
Distance from provincial center, mean, km	28.32	28.48	24.66	23.67	26.67	…
Features of facility, %						
Reception/registration room available	71	89	88	81	83	<.001
Waiting room available	66	77	80	75	75	.06
Separate waiting room for women available	54	72	72	67	66	.006
Heating available in winter	57	74	77	79	71	.001
EPI services provided	61	95	97	98	87	<.001
Training for vaccinators (n = 389)						
Initial training	81	84	85	88	85	.80
Refresher training	69	83	83	86	81	.16

Abbreviations: BHCs, basic health centers; CHCs, comprehensive health centers; DHs, district hospitals; EPI, Expanded Programme on Immunization; HSCs, health subcenters.

^a^ *P* values based on χ^2^ tests.

Based on estimates derived from a variety of sources, including the United Nations, the World Health Organization, the United Nations Children’s Fund (UNICEF), and several nongovernmental organizations, the mean catchment area population (ie, population of the geographic area served by a particular health facility) was estimated at 23 000 (95% CI, 20 225–25 776). Facility-type-specific catchment area estimates ranged from 11 574 (95% CI, 8770–14 378) for HSCs to 42 321 (28 029–56 613) for DHs. Only 78% of participating facilities, however, believed that their RI target population estimates (ie, typically children aged <2 years within the geographic area served by a health facility, who are eligible for RI services) were accurate, and even fewer facilities (68%) reported that local head counts were conducted in their catchment area to determine the size of each target population.

### RI Services

#### Demographics, Staffing, and Training

Of the 447 participating facilities, 389 (87%) offered RI services (Table 1). A stratified analysis by facility type showed that 98% of DHs, 97% of CHCs and 95% of BHCs offered RI services, whereas only 61% of HSCs offered such services (*P* < .001). Of the 389 facilities providing RI services, 331 (85%) reported having separate rooms for vaccination service delivery. On average, participating facilities had 2 full-time vaccinators on staff; the median number of full-time vaccinators was 2 for each facility type except HSCs, for which it was 1. The median numbers of female full-time and ancillary vaccinators were 1 and 0, respectively. Eighty-five percent of facilities reported that their vaccinators had received initial training, and 81% reported that ≥1 staff member routinely involved in vaccination had received refresher training within 1 year of the survey. Seventy-two percent of facilities reported that ≥1 staff member involved in other aspects of clinical care had received training in vaccination within 1 year of the survey. No significant disparities were observed by facility type when comparing the adequacy and frequency of training activities.

#### Documentation, Forms, and Cards

Immunization registers, tally sheets, and immunization summary sheets were said to be available and fully completed in 92%, 90%, and 85% of facilities, respectively (Table 2), and another 7% of facilities had immunization registers and tally sheets that were not fully completed. Eleven percent of participating facilities had immunization summary sheets that were not fully completed. Immunization coverage charts were available and completed in 89% of facilities, and another 7% of facilities had coverage charts that were not fully completed. In approximately 3% of facilities, the immunization coverage charts were either not available or were said to be available but not seen at the time of the survey. HSCs again lagged behind other facility types in availability of complete immunization registers (78% vs >93%; *P* < .008), tally sheets (80% vs >86%; *P* < .004), summary sheets (74% vs >81%; *P* < .02), and coverage charts (78% vs >87%; *P* < .009).

**Table 2:** Key Immunization Service Delivery Metrics Among Respondents in a Survey of Health Facilities Providing the Basic Package of Health Services in Afghanistan, 2014

Variable	Respondents, %					*P* Value^a^
	HSCs (n = 74)	BHCs (n = 127)	CHCs (n = 137)	DHs (n = 51)	Overall (N = 389)	
Documentation (present and completed)						
Immunization registers	78	93	96	98	92	.008
Tally sheets	80	86	96	100	90	.004
Summary sheets	74	81	91	96	85	.02
Vaccination cards	77	87	96	96	89	.001
Coverage charts	78	87	93	98	89	.009
Cold-chain management						
Refrigerator available	74	98	97	100	94	<.001
Refrigerator working	72	96	92	98	90	<.001
Power source available	53	81	77	82	74	<.001
Thermometer working	70	91	92	92	88	<.001
Functional cold boxes	68	92	94	98	89	<.001
Functional ice packs	59	83	90	90	82	<.001

Abbreviations: BHCs, basic health centers; CHCs, comprehensive health centers; DHs, district hospitals; HSCs, health subcenters.

^a^ *P* values based on χ^2^ tests.

Vaccination cards were available and completed in 89% of facilities, and an additional 7% of facilities had vaccination cards that were not fully completed. HSCs (77%) were less likely to have vaccination cards than other facility types (>87%). When asked about the adequacy of the supply of immunization cards 30 days before the survey, 95% of facilities stated that supplies were adequate, and a similar proportion stated that they issued immunization cards to children commencing their immunization schedules. In 91% of facilities, cards were given to the parent(s) once a child began receiving immunizations, whereas 3% of facilities retained the card on site and 2% issued a copy to the parent while retaining a copy on site. Vaccination card issuing practices were either unspecified or unknown in the remaining 4% of survey respondents.

#### Vaccine Availability and Cold-Chain Management

**Figure 1.**
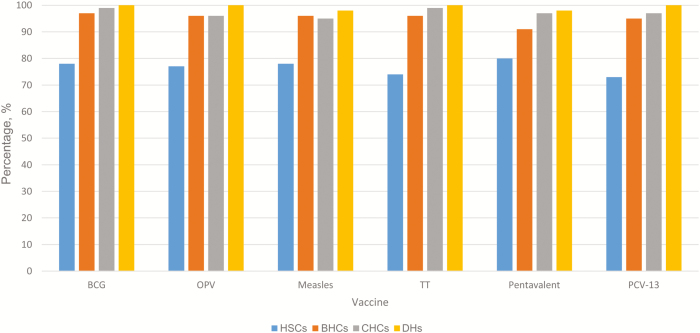
Percentages of respondents reporting an adequate vaccine supply in the 30 days before the 2014 Basic Package of Health Services survey in Afghanistan, by vaccine and facility type. BHCs, basic health centers (n = 127); CHCs, comprehensive health centers (n = 137); DHs, district hospitals (n = 51); HSCs, health subcenters (n = 74); OPV, oral poliovirus vaccine; PCV-13, pneumococcal conjugate vaccine; TT, tetanus toxoid.

Oral poliovirus (OPV), BCG, and measles vaccine stock were continuously available at ≥93% of participating facilities in the 30 days before the survey, but HSCs were less likely to report continued availability of stock relative to other types of facilities (OPV, 77% vs >96%; BCG, 78% vs >96%; and measles, 78% vs >94%; (Figure 1). All DHs were continuously stocked with OPV and BCG vaccine. Where not continuously available, the mean numbers of stock-out days (ie, days in which there were no supplies of a particular vaccine) were 4, 3, and 6 days for OPV, BCG, and measles vaccine, respectively. Approximately 1%–2% of facilities reported having vaccine vial monitors in stage 3 (expired vaccine) or did not have 1 of these vaccines in stock at the time of the survey.

Newer vaccines such as pneumococcal conjugate (PCV-13) and pentavalent (DPT/HBV/Hib) vaccine were also continuously available at approximately 92% of participating facilities in the 30 days before the survey, but HSCs were again less likely than other types of facilities to report continued availability of stock (PCV-13, 73% vs >95%; pentavalent vaccine, 80% vs >91%). All DHs were continuously stocked with PCV-13. In the few facilities in which PCV-13 and pentavalent vaccine were not continuously available, the mean number of stock-out days was approximately 6 days. As with other vaccines, approximately 1%–2% of facilities reported having vaccine vial monitors in stage 3 or did not have either vaccine in stock at the time of the survey.

Refrigerators for vaccine storage were available at 94% of participating facilities, though a slightly smaller percentage of facilities (90%) reported having a refrigerator in working condition **(Table 2**). Despite the high proportion of facilities with working refrigerators, HSCs were significantly less likely than other facility types to report having a refrigerator (74% vs >97%; *P* < .001) or one in working condition (72% vs >91%; *P* < .001). Only 74% of facilities reported having a functioning power source for the refrigerator. Main power sources included gas (68%), electricity (13%), and solar power (9%).

Vaccine cold-chain thermometers were present and working at 88% of participating facilities, but in fewer HSCs (70%) compared with other facility types (>91%). Temperature logs were kept twice daily in the 30 days before the survey at 73% of facilities; 12% of facilities completed their logs only once daily, whereas responses were unclear for all but 1% of the remaining facilities, which did not have logs. Cold boxes/vaccine carriers were present and functional in 89% of facilities, and 82% had ice packs available and in good condition. HSCs were less likely than other types of health facilities to have functioning cold boxes (68% vs >91%; *P* < .001) or ice packs in good condition (59% vs >82%; *P* < .001).

#### Service Delivery, Microplanning, and Social Mobilization

A variety of strategies were used in administering RI services to children. Among facilities that provided RI services, the primary strategy used among survey respondents was fixed-post (on-site) vaccination (40%). Fixed-post vaccination was often combined with outreach visits (21%) or both outreach and mobile team visits (24%). A small proportion of participating facilities administered services exclusively through outreach (11%) or mobile team (3%) visits. A majority of respondents (81%) complied with the WHO-recommended open-vial policy for multidose vials, although HSCs were less likely to be in compliance than other facility types (66% vs >84%, respectively). Where facilities complied with the open-vial policy, BCG and measles immunization sessions occurred on a mean of 10.29 (95% CI 9.28–11.29) and 10.30 (95% CI 9.29–11.30) days per month, respectively.

RI outreach microplans were available at 71% of participating facilities but were less likely to be available at HSCs (53%) than at other types of facilities (>71%). For facilities with RI microplans (n = 276), key components were identified as follows: immunization targets by settlement and session type (92%); maps showing catchment area boundaries, fixed/outreach, and mobile settlements (91%); identification of hard-to-reach and low-performance populations (81%); time schedule for immunization activities (92%); and scheduling of outreach settlements to receive ≥1 immunization visit per month (92%). No statistically significant differences were observed by facility type with respect to any of the key microplan components. A mean of 13 outreach sessions per facility were planned for the 3 months before the survey, with means ranging from 7 outreach sessions for HSCs to 16 for BHCs. However, only a mean of 11 outreach sessions per facility were implemented during the same time period, with means ranging from 6 outreach sessions for HSCs to 13 for BHCs.

Seventy-four percent of survey respondents had a focal person and an annual plan in place for social mobilization, public education, and communication at the health facility. Compared with other types of health facilities, HSCs were less likely to have a focal person (57% vs >72%; *P* < .002) or an annual plan for communication activities (54% vs >74%; *P* < .001). Thirty-eight percent of facilities reported having problems with implementing social mobilization activities, and 52% said they had none (10% of facilities could not be classified either way). Among facilities reporting problems with implementation (n = 149), the problems identified included staffing inadequacies/illness/strikes (28%), insufficient funding (14%), inadequate transportation or fuel (13%), insecurity (13%), and staff being preoccupied with other activities (9%).

## DISCUSSION

Previous assessments of the BPHS program have indicated that it has improved the delivery of essential health services to rural communities in Afghanistan [5]. This survey, limited in its scope to an assessment of RI services delivered through BPHS, corroborates findings from earlier assessments, but it also highlights several gaps and ongoing challenges in the implementation of the program. A majority of survey respondents (87%) provided RI services, and the availability of such services was even greater among the health facilities other than HSCs, which lagged behind other types of facilities in the provision of immunization services. Facilities had an average of 2 trained vaccinators, and a majority of respondents reported that staff had received initial training (>85%) and an annual refresher (>81%). The availability of essential vaccines, such as OPV, BCG, and measles vaccines, as well as new vaccines, such as pentavalent and pneumococcal vaccines, was high (>90%) at the different types of health facilities, except HSCs, where availability was typically <80%. Despite these positive findings, the spotlight in this report must remain on the gaps and deficiencies identified, in line with key objectives of the survey.

BPHS has affected RI services positively [6, 7] but the findings of this survey leave questions regarding the level of access to such services for persons living in rural Afghanistan. It is unclear whether substantial mean travel distances and times from provincial centers to health facilities are reflective of the travel burden on the target populations served by health facilities within their designated catchment areas. If these estimates indeed mirror the travel burden on the local populations, travel times could potentially limit access to such services, but even if they do not reflect travel times for persons within a catchment area, there could be implications for the delivery and availability of vaccine and cold-chain supplies. Given that fixed-post vaccination remains the primary strategy for immunization, it is imperative that the target population in a particular catchment area is able to access care within reasonable time (eg, within ≤30 minutes). Not only will this encourage greater levels of service utilization in the population [11–13], it will also enable easier follow-up of children who have begun their immunization series and may lead to fewer dropouts from the system. Moreover, the absence of waiting areas and the lack of heating during winter at about a quarter of participating facilities could discourage return visits by parents and compromise the likelihood that a child will complete his or her immunization series.

Staffing inadequacies at the HSC level, which averaged 1 vaccinator compared with 2 for other types of facilities, may hamper the ability to deliver RI services. Furthermore, unlike other facility types which had an average of 1 trained female vaccinator, most HSCs had none. This could hinder compliance with immunization, especially among women of childbearing age, given cultural sensitivities [5, 14]. Only DHs reported having a mean of ≥1 ancillary staff member trained in vaccination, implying limited preparedness for unforeseen circumstances (eg, illness of the vaccinator).

Although vaccine supplies were generally available at >90% at all levels of facilities except HSCs, the quality of storage and cold-chain management raised concerns. A majority of facilities reported having a refrigerator in working condition, but approximately one-quarter of respondents lacked a source of power. Gas was the main source of power, with electricity, solar power, and kerosene much less widely used alternatives. Whereas high proportions of DHs, CHCs, and BHCs had basic cold-chain equipment, such as thermometers, cold boxes, and ice packs, <70% of HSCs reported having an adequate amount of such supplies. This is worrisome, given how critical proper cold-chain management is to maintaining vaccine quality and potency [15, 16].

Compliance with recommended microplanning activities, which have been demonstrated to be of vital importance to immunization services [17, 18], was suboptimal. Only 71% of respondents had microplans in place, but among such facilities, a majority (>90%) met key requirements, such as outlining targets, catchment areas, planned immunization sessions and outreach schedules. Outreach sessions were, on average, planned to occur weekly, except for HSCs, where they were planned on a fortnightly basis. There was a small gap between planned and implemented immunization outreach sessions. The longer intervals between outreach sessions at the HSC level, probably imposed by staffing and travel constraints, should be further investigated and addressed in light of smaller, and thus more manageable, target populations in their catchment areas. Communication and social mobilization activities were hampered by the absence of a designated focal person at several facilities, because only 74% of respondents (<60% of HSCs) had a designee for such activities. Thirty-eight percent of facilities reported problems with implementation of planned activities, chief among which was inadequate staffing. Other key problems included insufficient funding, lack of transportation, and insecurity.

This report highlights the need to address critical deficiencies in the provision of immunization services through the BPHS program, especially at the HSC level, the primary level of service delivery among survey respondents. Access to immunization services may be improved by increasing the frequency and regularity of outreach sessions, especially for those who live at considerable distances and require significant travel times to obtain such services at fixed posts. Such outreach sessions should be based on detailed microplanning and appropriate communication strategies adapted to the local context. To accomplish this, staffing inadequacies must be addressed by ensuring that vaccinators as well as social mobilizers are hired, trained, and then assigned to health centers in numbers commensurate to the need in specific catchment areas. Infrastructural deficits, such as the lack of regular power supply and cold-chain equipment, should be prioritized as an area requiring urgent intervention at the HSC level. 

Ultimately, although the findings presented in this report indicate that RI service delivery remains a challenge, they also provide an opportunity for action to improve access and the quality of services available to children in Afghanistan. Given the critical role of RI in achieving the eradication of polio, investments by way of Global Polio Eradication Initiative funding to address gaps in RI service delivery through BPHS will fit within the overall strategy of the initiative and enhance the prospects for eliminating poliovirus from Afghanistan.

## Notes

***Acknowledgments.*** We acknowledge the contributions of all collaborators on this project, including staff of the Afghan Ministry of Public Health, the US CDC, the World Health Organization. and Apex Consulting, who participated in the design of the survey and subsequent implementation of the project. Specifically, we acknowledge the contributions of Diane Simpson, MD of the CDC and John Armstrong Stoney of the Center for Program Evaluation at the Melbourne Graduate School of Education, Australia, both of whom supported the development of the study protocol.

***Financial support.*** This work was supported by the CDC.

***Supplement sponsorship.*** This work is part of a supplement coordinated by the Task Force for Global Health with funding provided by The Bill and Melinda Gates Foundation and the Centers for Disease Control and Prevention.

***Potential conflicts of interest.*** All authors: No reported conflicts.
